# Determination of Chromium(III) and Chromium(VI) by Ammonium Pyrrolidine Dithiocarbamate-Methyl Isobutyl Ketone-Furnace Atomic Absorption Spectrometry

**DOI:** 10.6028/jres.093.055

**Published:** 1988-06-01

**Authors:** K. S. Subramanian

**Affiliations:** Environmental Health Directorate, Health and Welfare Canada, Tunney’s Pasture, Ottawa, Ontario, K1A 0L2, Canada

The determination of Cr(III) and Cr(VI) in environmental and biological systems is of interest because the toxicity of this metal depends on its oxidation state. Cr(III) is essential to mammals, while Cr(VI) is toxic. In addition to its existence in the two main oxidation states of +3 and +6, Cr occurs in the aquatic environment at ⩽ ng/mL levels. Therefore, some form of preliminary separation and preconcentration is required to determine the low levels of individual Cr species by sensitive analytical techniques, such as graphite furnace atomic absorption spectrometry (GFAAS) [1,2]. This paper explores the feasibility of directly complexing both Cr(III) and Cr(VI) by ammonium pyrrolidine dithiocarbamate (APDC) at room temperature, extracting these subsequently into methyl isobutyl ketone (MIBK) and determining the individual Cr species in the MIBK phase by GFAAS.

A Perkin-Elmer Model 603 atomic absorption spectrometer equipped with a Perkin-Elmer HGA500 graphite furnace, an AS-1 autosampler incorporating a 10-*μ*L pump, a PRS-10 printer and a Perkin-Elmer hollow cathode lamp operated at 25 mA at the Cr resonance line of 357.9 nm (spectral bandpass, 0.7 nm) were used for the determination of Cr. Argon served as the purge gas and its flow was interrupted during atomization.

The solution parameters studied include: pH of the aqueous phase, concentration of APDC, concentration of potassium hydrogen phthalate (PHP buffer, and length of time needed for complete extraction.

Complete transfer from aqueous to MIBK phase occurred in a single extraction at pH 2.5–4.0 for Cr(III) and pH 2.5–5.0 for Cr(VI) at final PHP and APDC levels of 0.1% and 1.2%, respectively. Thus simultaneous and quantitative extraction of Cr(III) and Cr(VI) were possible at pH 2.5–4.0. Also, Cr(VI) could be selectively and quantitatively extracted at pH 2.5–4.0, but at final APDC and PHP concentrations of 0.2% and 1%, respectively. In the case of Cr(III), the optimum APDC-to-metal and PHP-to-metal concentration ratios were ⩾2.4×10^4^ and 0.8×10^4^ to 4.0×10^4^, respectively (final concentrations: Cr(III), 50 ng/mL; APDC, 1.2%; PHP, 0.04–0.2%). Above 0.8% PHP, Cr(III) was not extracted whereas Cr(VI) was extracted. Thus selective extraction of Cr(VI) occurred when the APDC-to-metal and PHP-to-metal ratios were ⩾4×10^4^ and ⩾2×10^5^, respectively (final concentrations: Cr(VI), 50 ng/mL; APDC, 0.2%; PHP, 1%). Extractions of Cr(III) and Cr(VI) were complete on shaking for 20 min. and 10 min. respectively, at pH 3.5 and 22 °C. Both the Cr(III) and Cr(VI) chelates were stable for at least 30 days when the MIBK phases were transferred to dry centrifuge tubes soon after extraction. In summary, these studies showed that, by careful optimization of the concentrations of APDC, PHP, pH and extraction time, the APDC-MIBK system could be used to: (i) selectively separate Cr(VI) from Cr(III); and (ii) simultaneously extract Cr(III) and Cr(VI) at room temperature without the need to oxidize Cr(III) to Cr(VI).

The sensitivity (concentration at 0.0044 absorbance), detection limit (3 SD of blank) and linear range for both Cr(III) and Cr(VI) were identical and found to be (ng/mL): 0.2, 0.3 and 0–50, respectively, in the MIBK phase. The values in the aqueous phase depend on the phase volume ratio. A concentration factor of up to 20 was possible with MIBK. At 20-fold concentration the above values will be (ng/mL): 0.01, 0.02 and 0–2.5, respectively. Thus the method is sufficiently sensitive to permit determination of background levels of Cr(III) and Cr(VI) in unpolluted natural and drinking water samples. The precision, expressed as the coefficient of variation (CV), at 5, 10 and 20 times the detection limit of Cr was 33.3, 12.6 and 8.1, respectively. The reliability of the proposed procedure was assessed by analyzing the NBS multielement water standards SRM 1643a and SRM 1643b, and the National Research Council of Canada (NRCC) river water standard, SLRS-1. Both the NBS and the NRCC specify certified values only for total Cr. The observed values for total Cr of 18.1±0.6 (SD), 17.8±0.3, and 0.22±0.05 ng/mL for SRM 1643a, SRM 1643b, and SLRS-1, respectively, compare favorably with the corresponding certified values of 17.3±2.0, 18.9±0.4 and 0.36±0.04 ng/mL. Furthermore, our analysis revealed Cr(III) to be the dominant species in the NBS standards as confirmed by the good agreement for Cr(III) by the APDC-MIBK extraction procedure and an ion exchange procedure based on the use of a Bio-Rad SM-7 polyacrylic ester resin [1]. Thus the solvent extraction values for Cr(III) in SRM 1643a and SRM 1643b were (ng/mL): 16.0 and 16.4, respectively, while the corresponding ion exchange values were 14.6±0.5 and 17.9±0.5 ng/mL. The reliability of the APDC-MIBK-GFAAS procedure was also ascertained by doing recovery studies. The average percent recovery obtained for the addition of Cr(III), Cr(VI) and Cr(III)+Cr(VI) spikes to tap, well, treated and river water samples were quantitative.

No interferences were found from Ca, Mg, Na, K and a number of trace elements at levels exceeding those usually found in most fresh and drinking water systems. The humic acid concentration in the solution to be extracted for Cr(III) should be ⩽2 mg/mL. In the case of Cr(VI), up to 40 mg/L humic acid can be tolerated.

The method was applied to the determination of Cr(III) and Cr(VI) in a number of water samples according to the procedures outlined below.

- Determination of Cr(III)+Cr(VI). To 25 mL of sample, add 0.3 mL of 8% PHP, adjust pH to 3.5, and add 3 mL of 10% APDC and 5 mL of PHP-saturated MIBK. Extract for 20 min. Determine Cr(III)+Cr(VI) in the MIBK layer.
- Determination of Cr(VI). Proceed as above except for the addition of 3 mL of 8% PHP and 3 mL of 2% APDC and for the extraction time of 10 mm.
- Determination of Cr(III). The value of Cr(III) is obtained as the difference between Cr(III) +Cr(VI) and Cr(VI). The concentration of Cr(III) is also determined by the SM-7 ion exchange procedure [1].

In all the above cases, the Cr atomic absorption signal was measured by using the dry/atomize program of 900 °C–30 s (ramp)–30 s (hold)/2500 °C–0 s (ramp)–6 s (hold).
